# Control of the Inflammatory Response Mechanisms Mediated by Natural and Induced Regulatory T-Cells in HCV-, HTLV-1-, and EBV-Associated Cancers

**DOI:** 10.1155/2014/564296

**Published:** 2014-11-30

**Authors:** Laurissa Ouaguia, Dhafer Mrizak, Sarah Renaud, Olivier Moralès, Nadira Delhem

**Affiliations:** ^1^CNRS UMR 8161, Institut de Biologie de Lille, Universit Lille-Nord de France, SIRIC ONCOLille, Institut Pasteur de Lille, IFR142, 1 rue du Professeur Calmette, 59021 Lille Cedex, France; ^2^UFR de Biologie, Université de Lille 1, Cité Scientifique, Bâtiment SN3, 59655 Villeneuve d'Ascq Cedex, France

## Abstract

Virus infections are involved in chronic inflammation and, in some cases, cancer development. Although a viral infection activates the immune system's response that eradicates the pathogen mainly through inflammatory mechanisms, it is now recognized that this inflammatory condition is also favorable to the development of tumors. Indeed, it is well described that viruses, such as hepatitis C virus (HCV), Epstein Barr virus (EBV), human papillomavirus (HPV) or human T-cell lymphotropic virus type-1 (HTLV-1), are important risk factors for tumor malignancies. The inflammatory response is a fundamental immune mechanism which involves several molecular and cellular components consisting of cytokines and chemokines that are released by various proinflammatory cells. In parallel to this process, some endogenous recruited components release anti-inflammatory mediators to restore homeostasis. The development of tools and strategies using viruses to hijack the immune response is mostly linked to the presence of regulatory T-cells (Treg) that can inhibit inflammation and antiviral responses of other effector cells. In this review, we will focus on current understanding of the role of natural and induced Treg in the control and the resolution of inflammatory response in HCV-, HTLV-1-, and EBV-associated cancers.

## 1. Introduction

Cancer is a serious disease and a leading cause of death in the world. It can develop after a bacterial, parasitic, or viral infection [1]. Viruses and bacteria can cause chronic inflammation and are thought to contribute to more than 1.2 million cases of infection-related disease per year [2, 3]. For example, hepatitis C virus (HCV), Epstein Barr virus (EBV), human papillomavirus (HPV), Kaposi sarcoma-associated herpes virus (KSHV), and human T-cell lymphotropic virus type-1 (HTLV-1) are important risk factors for malignancies such as hepatocellular carcinoma (HCC), nasopharyngeal carcinoma (NPC), cervical cancer, Kaposi sarcoma (KS), and Adult T-cell leukemia (ATL), respectively [4–6]. Such viruses act through inflammation-related mechanisms in addition to the inhibition of tumor suppressive genes [7]. It has been shown that some viral cellular transformations happen when the virus genome interacts with the DNA of the host cell. Those viruses are called oncogenic viruses, that is, human tumor-viruses [5, 8]. Thereby, this results into uncontrolled cell growth that occurs with the invasion of surrounding tissues and the spread of malignant cells. The viral infection or the presence of a tumor cell activates the immune system's response involving a wide range of components that are resumed under two general responses: the “innate immune response” involving mainly neutrophils, monocytes, and dendritic cells and the “adaptive immune response” which implies B and T lymphocytes. The innate response provides the first line of defense against invading pathogens. It leads to the halt of the initial spread of infection but also activates the adaptive immunity and other secondary host defense mechanisms [9, 10]. The adaptive immune response is mediated by the B and T lymphocytes [11, 12]. The major goal of this immune response is to eradicate the pathogen mainly through inflammation mechanisms [13]. Indeed, inflammation and immunity probably affect different stages of cancer development with inflammation and innate immunity most commonly exerting protumorigenic effects while adaptive immunity potentially exerts antitumorigenic effects [14]. However, it is now recognized that the inflammatory condition is favorable to the development of tumors [2, 15]. Nevertheless, the inflammation's role in a wide variety of diseases such as cancer has just been evaluated [14, 16]. While acute inflammation seems to be a part of the antipathogenic response, chronic inflammation can also lead to cancer [16].

The inflammatory response is a fundamental immune mechanism known to be a localized protective reaction of tissue against irritation, infection, injury, allergy, and tumors. Inflammation is characterized by redness, pain, and thickness. This process involves several molecular and cellular components consisting of lipid inflammatory mediators (leukotriene, prostaglandin, etc.) and cytokines (IFN*γ*, TNF*α*, IL2, IL12, etc.), as well as chemokines (CCL20, CXCL10, CCL22, CCL17, etc.), that are released by various proinflammatory cells such as T helper (Th)1, Th17, Th9, Th22, monocytes, macrophages, dendritic cells, and cancer cells themselves [6, 13, 15, 17–19]. The release of proinflammatory factors by such cells leads to the recruitment of numerous proinflammatory cells to the infection site [6]. At the same time, a number of other cells are also recruited in order to control the inflammation and to avoid chronic inflammation, a characteristic in many chronic diseases such as cancer. Thereby, some endogenous recruited components would release anti-inflammatory and proresolving mediators to restore homeostasis. The HCV antigens will activate the humoral and cellular immune responses. The development of mechanisms and strategies by the immune system to restore immune homeostasis is mostly linked to the presence of Th2 but primarily to regulatory T-cells that can inhibit inflammation and the responses of others effectors cells [11, 19, 20].

Regulatory T-cells (Treg) are specialized subsets of the adaptive immune response which are able to recognize antigen peptides presented by MHC classes I and II via their TCR. Treg cells suppress the autoreactive T-cells, reduce inflammation, induce tolerance, and modulate the immune response of the host in the context of autoimmune pathologies, allergies, or virus-induced cancers [10, 19]. The “suppressor T-cells” hypothesis was enunciated by Vadas and his collaborators in 1976 but the common term “regulatory T-cells” appeared for the first time with Sakaguchi in 1995 [21, 22]. In Humans, the lack of Treg cells is associated with a multisystemic autoimmune disease known as IPEX (i.e., immunodysregulation, polyendocrinopathy, and enteropaty, X-linked syndrome) [23]. Therefore, Treg cells seem to be major inhibitors of autoimmune diseases.

For a decade, Treg have been widely described to be involved in cancer. Indeed, most of Treg cells are chemoattracted to tumor tissues where they proliferate locally and differentiate into different Treg cell subpopulations that strongly suppress the activation and the expansion of tumor-antigen-specific effector T-cells [18, 20]. CD4+ regulatory T-cells refer to the major Treg subset that has been described for decades. Depending on their origin and the expression of cell surface markers, we can distinguish 2 different types of CD4+ regulatory T-cells in humans. Briefly, the first type is a natural regulatory T-cells (nTreg) deriving from thymus.* In vitro*, those cells are anergic and are characterized by a high expression of the transcription factor forkhead box 3 (FOXP3), a constitutive cell surface expression of CD25 (the interleukin-2 receptor alpha-chain), and both cell surface and cytoplasmic expression of CTLA4 (the coinhibitory receptor cytotoxic T lymphocyte antigen 4) [11, 19, 23]. Since their description, several extra molecules have been put forward as Treg markers. This includes GITR (glucocorticoid-induced TNF receptor family related protein) [24], PD-1 (programmed death 1), LAG3 (lymphocytes activation gene 3) [20, 25], HLA-DR [26], CD45RA/CD45RO [27], CD62L (L-selectin), CD44, CD28 (costimulatory molecule), CCR7 (chemokine (C-C motif) receptor 7), CXCR4, OX40 (CD134), Folate receptor-4 [28], and CD39 [29]. Natural Treg cells were also characterized by a lack or reduced expression of CD127 (the interleukin-7 receptor alpha-chain) [30]. It has been demonstrated that nTreg exert their suppressive functions either through cell-to-cell contact-dependent mechanisms via membrane-bound molecules or through the secretion of immunosuppressive cytokines [31]. For example, the high CD25 expression on nTreg cells gives it the ability to consume large concentrations of the proliferative cytokine IL2 and thereby, nTreg may inhibit the proliferation and induce apoptosis of surrounding T-cells. Moreover, CTLA4 interacts with CD80/CD86 and thus inhibits the T-cell costimulation. In addition, nTreg cells produce TGF*β* (transforming growth factor beta) and IL10 (interleukin 10) to reduce antigen presentation, to prevent antigen presenting cells (APCs) maturation and to induce cell cycle arrest. By CD39 and CD73 expression, nTreg cells also inhibit the ATP metabolism and thus promote cell cycle arrest [31]. The second regulatory CD4+ T-cell population consists of induced or adaptive Treg cells that can be divided into 3 subsets. First, we distinguish Tr1 or T regulatory type 1 cells which secrete vast quantities of IL10 associated with a mild secretion of TGF*β*. This subset is characterized by a CD4, CD18, CD49b, LAG3, and GATA-3 expression, lack of FOXP3, and relative CD25 expression [32]. Tr1 are anergic* in vitro* and they suppress the cell proliferation through their IL10 production [33]. Secondly, we distinguish Th3 or T helper 3 that can be characterized by a mild production of TGF*β*, IL4, and IL10. Due to TGF*β* presence, naïve CD4+ T-cells can differentiate into Th3 cells which possess an important role in negating autoimmune reactions and promoting oral tolerance. There is some evidence suggesting that Th3 cells can express some nTreg molecules such as CD25, FOXP3, and CTLA4 [27, 34]. The third subset of iTreg, and the less studied, is iTR35 or IL35-producing-CD4+ T-cells. Recently, it has been shown that IL35, made up of two subunits IL12p35 and Ebi3, may induce the emergence of regulatory T-cells that mediate the suppression in a IL35-dependent manner [11, 19, 25, 35–37]. In general, induced regulatory CD4+ T-cells result from the peripheral differentiation of effector T-cells according to the nature of the antigen or to the cytokinic environment [11, 36, 38]. As such, they represent the major subset of Treg present in cancer [39].

Other T-cell populations that have been described to exhibit regulatory functions are composed of CD8+ Treg [27, 40] and CD3+CD4−CD8− Treg cells [41]. These cells subsequently decrease the priming of cytotoxic CD8+ T-cells, regulate the immune response by cell-to-cell mechanisms, and promote the development of tolerance [42]. Some CD8+ Treg subsets are characterized by the expression of numerous markers like CD25, CTLA4, FOXp3, HLA-DR, CD28, LAG3, and GITR. Their suppressive function is exerted in a cell-to-cell contact-dependent manner like that observed in nTreg. Some CD8+ Treg cells may have a thymic origin while others possess a peripheral one. Besides, there are also natural killer regulatory T-cells (NKT Treg) generated from the thymus that can be CD4+, CD8+, CD4−CD8−, or CD4+CD8+. The latter secrete IL4, TGF*β*, and IL10 to induce APCs cytotoxicity by a cell-to-cell contact-dependent mechanism [43]. Another small subset of Treg is gamma delta T regulatory cells associated with the mucosal tolerance in Human [44].

To sum up their functions, Treg cells (natural and induced) provide an important mechanism for the maintenance of central and peripheral self-tolerance [11, 20]. Treg exert their suppressive abilities to inhibit the effector's cytotoxic antiviral and antitumoral responses in the tumor cells environment [19]. By such skills, Treg favor the establishment and persistence of many pathogens like viruses and could then promote the progression of virus-related cancers [45].

Until today, the role of Treg in cancer development and progression is not clear. Indeed, Treg appear to play a dual role in cancer. Earlier evidence have suggested that Treg cells accumulate in tumors and peripheral blood of cancer patients and through suppression of antitumor immune responses promote tumor growth such as in hepatocellular carcinoma [46, 47], nasopharyngeal carcinoma [48], cervical cancer, Kaposi sarcoma, ovarian cancer, and melanoma [49]. This is the reason why Treg cells are often associated with a bad prognosis in cancer [36, 37, 46, 47, 50, 51]. However, other evidences indicate that in certain cancers, such as colorectal carcinoma [38], Hodgkin lymphoma, and estrogen receptor (ER) negative breast cancer, Treg suppress the pathogenic agent and thus are of good prognosis [52]. Such a difference implies that the clinical and prognostic outcomes of Treg in cancer depends on environmental factors including infectious agents, tumor-derived products, and locally-produced cytokines, which shape the nature of immune responses, including Treg generation, recruitment, and function [39, 53].

In this review, we will focus on current understandings of the role of natural and induced Treg in the control and resolution of inflammatory response in HCV-, HTLV-1-, and EBV-associated-cancers.

## 2. Role of Treg Cells in the Inflammation Associated with HCV-Induced Hepatocellular Carcinoma

Based on the data obtained from WHO in 2008, hepatocellular carcinoma (HCC) represents the fifth cause of death in men and the seventh in women worldwide [1]. In general, HCC is the third most common cause of cancer related death [54]. The incidence of HCC correlates with the incidence of hepatitis C and B infections [55]. HCC is one of the most aggressive cancers with an overall survival rate of 10% in 5 years, even in developed countries [56]. HCC is a heterogeneous tumor of varied etiologies. The hepatocarcinogenesis is an asymptomatic and complex multistep process developing, in most cases, after decades of chronic liver disease, leading to the progressive accumulation of genetic and epigenetic alterations such as oncogenic activation, inactivation of tumor suppressive genes, and activation of cell proliferation pathways, release of angiogenic and growth factors that result in the malignant transformation of a healthy liver cells [57]. Nevertheless, it is still unclear at what stage the cumulative disorders become irreversible. The only effective approaches for patients with HCC are resection or liver transplantation. It has been reported that HCV infection increases the risk of HCC through the development of hepatic fibrosis and cirrhosis [58, 59]. Indeed, HCV infection is a persistent disease with a majority of patients remaining asymptomatic. However, in some individuals, the disease progresses at a variable rate from active inflammation to fibrosis and possibly cirrhosis. The onset of cirrhosis precedes the appearance of liver-related complication such as the development of hepatocellular carcinoma [5].

Based on a recent study, we know that hepatocarcinogenesis is promoted by an accelerated cellular turnover induced by chronic tissue damage and permanent cell regeneration in a context of chronic inflammation and sometimes after a viral infection [60]. Following HCV and HBV infection, human hepatocytes express and present the viral antigens to CD4+ T and CD8+ T-cells which are able to clear the virus by noncytolytic and cytolytic effector functions [61]. There is growing consensus that CD8+ T response plays a central role in the inhibition of the viral replication mediated by cytokines and direct killing of infected hepatocytes [62]. The CD4+ activation may direct Th1 response with the secretion of IL2, TNF*α*, and interferon (IFN) [9]. In addition, HCV CD4+ T stimulation may also induce Th2 cells which secrete IL4, IL5, IL10, and IL13 [13]. More recently, a third population, Th17, has been described to be involved in hepatic chronic inflammation following HCV and HBV infection [4, 63]. Th17 are induced proinflammatory CD4+ T-cells characterized by their ability to secrete specific inflammatory cytokines such as IL17, IL21, IL22, IL6, and IL26. In HCV-derived HCC, numerous Th17 cells have been described within the tumor [64]. Th17 exhibits either proinflammatory or protumoral functions [65] and a high proportion of Th17 cells have been described in advanced tumoral stages [66]. Several studies have mentioned that a large number of HCC patients exhibit those specific inflammatory immune responses. In fact, the HCV-HBV-associated HCC microenvironment is colonized by infiltrated inflammatory immune cells such as macrophages, NK, B, and T lymphocytes [67]. It has been reported that tumor cells release a number of cytokines and chemokines factors [9, 67, 68]. The production of these inflammatory factors may be linked to the viral hepatocarcinogenesis and it was reported that these inflammatory cytokines are involved in the activation of the NF*κ*B pathway [69–72]. Nevertheless, in most patients the tumor progresses despite this immune response. This strongly suggests that HCC escapes from the immune response and this might probably be due to the HCC suppressive microenvironment which is known to play a key role in tumor progression. In fact, liver is a special organ with a unique regulatory microenvironment with certain liver sinusoidal cell populations that actively promote the induction of tolerance rather than immunity [46, 47, 62]. Numerous immune regulatory mediators such as IL10 and TGF*β* are secreted by Kupffer cells and stellate cells towards innate and adaptive immune inflammation [62]. In addition, liver cells are also characterized by the constitutive expression of tryptophan-2,3-disoxygenase that convert tryptophan into regulatory components as well as the indoleamine-2,3-disoxygenase (IDO) which is induced during inflammation via interferon signaling and is implicated in the production of immune regulatory components [73]. Another mechanism in inflammation control is the constitutive expression of arginase by hepatic cells, an enzyme which inhibits the lymphocytes proliferation [74]. Moreover, in some patients, tolerogenic hepatic cells may prime BCL-2 interacting mediator of cell death (BIM) which can induced T-cell apoptosis [70].

Unlike other Viruses, HCV does not cause a systemic ablation of the immune system but is rather associated with a form of specific tolerance much like the immune response to HCV; it is stopped and is unable to eliminate the virus [75]. Very interestingly, it has been described that infected hepatic cells overexpress programmed death ligand 1 (PDL1) which is the immunosuppressive ligand for PD1. Besides, HCV hijacks specific effector T-cells, so they express immunomodulatory factors such as CTLA4, Tim3, and 2B4 (CD244) followed by the decrease of their IFN secretion, mainly by iTreg [76, 77]. The cytokine's secretion controls the immune and inflammatory environment to either favor antitumor immunity or enhance tumor progression through IL10, TGF*β*, IL6, IL17, or IL23 [72]. The immune suppressive network within the HCC microenvironment is probably one of the major obstacles to the success of cancer immunotherapy.

Through their own proteins, some viruses such as HCV could also inhibit the immune inflammation. In the past, several papers have demonstrated that HCV-Core and NS5A proteins are involved in immune suppression by inducing effector T-cells apoptosis [78–80]. Furthermore, E1, E2, and NS3/4 are also involved in the modulation of endoplasmic reticulum stress, modulation of the PKR pathway, and cell transformation, respectively [81]. All these HCV features give the virus the ability to regulate the immune response and thus favor virus persistence. HCV infection appears to interact closely with the immune cells by infecting lymphoid cells [82].

Another explanation of this phenomenon could be the differentiation and/or recruitment of intrahepatic regulatory T-cells which is probably one of the key factors of the disease progression. It has been shown that natural regulatory CD4+ T-cells are highly recruited within the HCC where they secrete IL10 and TGF*β* to suppress the antitumor response and promote the tumor growth [62, 67, 83]. These Treg cells suppress the proliferation and cytokine secretion of HBV- and HCV-specific effectors T-cells in a cell-to-cell contact-dependent manner [62]. More interestingly, it has been described that CD4+CD25+FOXP3+ Treg cells were highly enriched with TIL (tumor infiltrating lymphocytes) in advanced-stage HCC-patients [84, 85]. These HCC-Treg cells may suppress effector cells' proliferation, activation, cytokine secretion, degranulation, and cytotoxic compounds production [84, 85].* In vitro* experiments also revealed that isolated HCC-infiltrating CD4+CD25+FOXP3+ regulatory T-cells can directly delete the cytotoxic function and the IFN*γ* secretion of some effector TIL in a TGF*β*- and IL10-dependent manner [62, 86]. Moreover, it has been shown that the CD4+CD25+ T-cells fraction isolated from peripheral blood mononuclear cells of infected patients might suppress the virus-specific CD8+ T-cells proliferation, the antiviral immune response, and a depletion of this fraction resulted in increased IFN*γ* production [85, 87]. Interestingly, an increased quantity of circulating nTreg and FOXP3+ Treg cells was associated with high mortality and poor survival time of HCC patients [46, 84]. Treg cells can also suppress the proliferation and cytokines production of HBV- and HCV-specific CD8+ T effector cells through IL10 and TGF*β* cytokine production [62, 84]. Indeed, some results showed that a proportion of FOXP3+CD4+ Treg cells may secrete IL10, a cytokine which promotes HCV persistence through its suppressive function on effector T-cells. Moreover, others reveal HCV-specific IL10 producing type 1 Treg (Tr1), found in persistent HCV infections [88, 89]. In addition, an upregulation of Th3 cells and Tr1 cells has been described in early chronic HCV infection which is characteristic of the immune modulation [90]. HCV is remarkable at disrupting human immune response to favor disease development until cancer. Indeed, recent studies revealed that HCV-infected hepatocytes drive nTreg development through the Tim3/Galectin-9 pathway. This pathway has also been shown to play a pivotal role in suppressing antiviral effector T-cells that are essential for viral clearance [91, 92]. Otherwise, it has been mentioned that CD25−FOXP3− Treg cells that possess suppressive abilities are increased in HCC-patients [83]. Other data showed that CD4+ Treg cells may upregulate Tim-3 expression therewith IL10 and TGF*β* production; Tim3/galectin9 interactions have also been shown to negatively regulate effectors T-cells [91]. Furthermore, there is some evidence of antigen specific induction of CD4+Treg cells (iTreg) after HCV infection. Indeed, other studies have shown that HCV may drive conventional CD4+ T-cells conversion into CD25+FOXP3+ Treg cells. In addition, others show that HCV variants may simultaneously upregulate inhibitory IL10, TGF*β*+Th3, and IL10+Tr1 cells which promote the inhibition of the T-cell proliferation [90]. The induction of iTreg may be favored by locally increased levels of TGF*β* and IL10 produced by infected hepatic cells and/or by recruited Treg cells. These induced Treg could be classified into CD4+TGF*β*+Th3 and CD4+IL10+Tr1 cells which secrete the immunosuppressive cytokines TGF*β* and IL10, respectively, in order to inhibit the inflammatory response [83, 90]. Otherwise, we and others have also demonstrated that Treg cells appear to highly accumulate within the hepatic tumor region compared with the nontumor region [84]. Hence, Treg seem to closely interact with HCV and this may lead to an increase of their regulatory phenotype and function as well as increasing their ability to suppress the inflammatory immune response. Some studies have also mentioned that circulating Treg cells frequency was significantly increased and correlates with the disease progression in HCC patients [84].

In addition to CD4+Treg cells, there are also CD8+Treg cells present in persistent liver disease. In fact, FOXP3 expression was described on a fraction of CD8+ T-cells with a reduced ability to proliferate or secrete antiviral cytokines. These impaired CD8+ T-cells were associated with the coexpression of inhibitory receptors such as PD-1, CTLA4, TIM-3, and CD44. The CD8+ Treg cells appear to be involved in the inhibition of an efficient virus-specific T immune response [62, 93]. CD8+ Treg cells are known to produce high levels of IL10 and/or TGF*β* to suppress HCV-specific effector T-cells [94]. Taken together, the data strongly suggest that the function and frequency of regulatory T-cells have been shown to correlate in a proportionally inverse manner to the antiviral response. Therefore, the Treg cells are involved in the aggravation of liver disease, notably in the development of hepatocellular carcinoma [28].

## 3. Role of Treg Cells in the Inflammation Associated with HTLV-1-Induced Adult T-Cell Leukemia/Lymphoma

Adult T-cell leukemia/lymphoma (ATLL) is a human disease associated with the infection of the retrovirus human T-cell leukemia virus type 1 (HTLV-1) [95]. It has been estimated that around 20 million people are infected with HTLV-1 worldwide [96]. HTLV-1 infection is a direct cause of ATLL, which can, through induction of immunodeficiency, indirectly cause many other chronic inflammatory diseases such as monoclonal gammopathy, chronic renal failure, strongyloidiasis, and HAM/TSP (HTLV-1 associated myelopathy/tropical spastic paraparesis) [97–99]. In addition to the typical ATLL, HTLV-1 seems to be also associated with a Hodgkin-like ATLL disease, gastrointestinal tract tumor and leukemia [100, 101]. Leukemic cells can be infected by HTLV-1 as it possesses a proviral DNA which can integrate into the cell's DNA [100]. This proviral load may contribute to the development of HTLV-1-associated inflammatory conditions, since the number of circulating HTLV-1-infected T-cells in the peripheral blood is higher in patients with HAM/TSP than in asymptomatic HTLV-1-infected individuals [102].

HTLV-1 is the first human retrovirus that has been described [100]. It utilizes CD4+ T-cells as their main host and its pathogenicity may be due to an abnormal frequency and phenotype of CD4+ T-cells in infected patients [103]. Invasion by HTLV-1-infected T-cells, together with viral gene expression and cellular-signaling mechanisms, triggers a strong virus-specific immune response and increased proinflammatory cytokine production [6]. Cellular immune response has been implicated in the control of HTLV-1 infection as well as in the development of inflammatory alterations in patients [45]. Following HTLV-1 infection, the viral antigen set induces host immune responses including B-cells for antibody production, cytotoxic T lymphocytes (CD8+ T-cells), and T helper cells (CD4+ T-cells). Under specific conditions, CD4+ T-cells differentiate towards Th1, Th2, and Th17 [6]. These cells produce a variety of proinflammatory cytokines, chemokines, adhesions molecules, and proinflammatory enzymes involved in chronic inflammation which are reactive oxygen species (ROS); tumor necrosis factor alpha (TNF*α*); IL1, 6, 8, and 18; nuclear factor-kappa B (NF*κ*B); hypoxia-inducible factor (HIF); IFN*γ*; cyclooxygenase (COX); and so forth [16, 102]. Other results showed that ATLL cells could produce TNF*α* to mediate inflammation and the TNF*α* polymorphism was associated with increased susceptibility to develop ATLL in HTLV-1 carriers [104]. TNF*α* will activate the NF*κ*B pathway which acts as a crossroad for the secretion of inflammatory factors in cancer [16]. NF*κ*B transcription factor plays a key role in the host's antiviral response involving both the innate and adaptive arms of the immune response. Interestingly, HTLV-1 possibly exploits NF*κ*B for its replicative, widespread, and pathogenic functions [104, 105]. HTLV-1 encodes for several structural viral proteins as well as several regulatory proteins like Tax protein. Moreover, it has been described that the Tax transforming protein encoded by HTLV-1 persistently activates and binds to the TCR/NF*κ*B axis [106]. Thus, HTLV-1 Taxis one of the exogenous retrovirus genes responsible for immune dysregulation [6]. Otherwise, HTLV-1 infection can induce the overtranscription of IRF4 (interferon regulatory factor 4) which can block the caspase 3 activity and promote B-cell integration cluster (BIC). IRF4 is an oncogenic factor favoring tumor development [90]. Hence, IRF4 and BIC could play key roles in HTLV-1 tumorigenesis up to ATLL [90].

Many viruses are able to develop suitable strategies for modifying apoptosis in virus-infected cells or/and cancer cells. As the induction of apoptosis may result in the viral elimination, inhibition of apoptosis may result, in turn, in cell transformation and viral persistence. Interestingly, it has been shown that ATLL cells exhibit Treg cell-like suppressor activity [107]. HTLV-1 infection induces the expression of these regulatory proteins in order to favor viral cell latency, host cell proliferation, and persistent infection [103]. For example, through the inhibition of Th1 cytokine production, the HTLV-1-HBZ protein can impair cell-mediated immunity and promote immunodeficiency. In addition, HTLV-1 Tax could reduce the regulatory FOXP3 and cell surface markers CD38, CD62L, CTLA4, and GITR expression. Other studies reported that HTLV-1 Tax could induce the antiapoptotic protein Bcl-2 to enhance the survival of infected or ATLL cells [108].

ATLL is an aggressive malignancy in which neoplastic cells usually exhibit a CD4+CD25+FOXP3+ phenotype [109, 110]. It is known that regulatory FOXP3+ T-cells are characterized by the expression of the CD4 and CD25 markers and therefore, it has been hypothesized that ATL cells may be derived from Treg cells [11, 111]. In the early phase of ATLL, there is a period of autocrine growth of the leukemic CD4+ T-cells with the expression of IL2 and its functional receptors. Over time, IL2 production is lost, although the cell surface expression of CD25 persists. Nowadays, regulatory CD4+CD25+ T-cell population is known as the main reservoir of HTLV-1 in HAM/TSP patients with more than 90% of Treg containing HTLV-1 proviral DNA and a higher expression of HTLV-1 Tax protein [112, 113]. Indeed, HTLV-1 usually infects regulatory CD4+FOXP3+ T-cells and this may facilitate persistent infection* in vivo*. This pattern could contribute to the pathogenesis of the virus-associated diseases [103]. In HTLV-1 infected patients, Treg cells may exert their suppressive function through the production of soluble factors acting at both long and short distances. Indeed, cytokines such as IL10 and TGF*β* have been related to the progression of the HTLV-1-related disease [114]. In addition, it has been shown that CD4+FOXP3+ Treg cells may also exert their regulatory effect on HTLV-1-specific CD8+ T-cells through a cell-to-cell contact-dependent manner with or without the involvement of cytokines [115]. Likewise, it has been reported that HTLV-1 infection increases the CTLA4 and GITR expression in Treg cells in order to increase the cell-to-cell suppressive mechanism [115]. For example, the CTLA4 molecule triggers the induction of IDO when it interacts with ligands in dendritic cells. ATLL cells exhibit immunosuppressive functions similar to Treg cells, and this may contribute to a clinically-observed cellular immunodeficiency in some patients [111]. Nevertheless, other studies reveal that some of these ATLL cells may lose their regulatory function with time [116]. Indeed, they described that TFG*β* signaling dysregulation and FOXP3 gene silencing could be involved in the pathogenesis of HAM/TSP. Hence, the viral pathogenesis could be improved by a loss of FOXP3 expression [115]. In addition, other results revealed that the level of FOXP3 expression was significantly decreased in CD4+CD25+ T-cells in HAM/TSP [113]. Based on the expression level of FOXP3 and CD45RA markers, we can distinguish three distinct subsets of CD4+ T-cells: (i) CD45RA^+^FOXP3^+^ resting Treg cells; (ii) CD45RA^−^FOXP3^high^ activated Treg cells; and (iii) CD45RA^−^FOXP3^low^ nonsuppressive Treg cells [117]. Several studies suggest that FOXP3+ATLL cells belong to these subpopulations meaning that ATLL cells may also fall into the group of nonsuppressive T-cells. This may explain the controversial outcomes of previous studies on the suppressive abilities of ATL cells [118]. Moreover, other studies revealed elevated frequencies of nTreg, associated with poor prognosis in patients with acute leukemia [119]. In addition, some studies reported that FOXP3 induces high levels of miR155 expression in Treg cells for a better suppressive function [120]. miR-155 is a regulator of innate immunity, hematopoiesis, and lymphocytes homeostasis [121]. Most CD4+CD25+ATLL cells express FOXP3 transcription factor. Recently, other results showed that HTVL-1 could modulate the frequency and phenotype of FOXP3+CD4+ regulatory T-cells in virus-infected individuals [103]. HTLV-1 infected T-cells produce CCL22 through Tax protein which selectively attracts Treg cells. This phenomenon contributes indirectly to the generation and the maintenance of HTLV-1 uninfected FOXP3+cells [122]. Thereby, HTLV-1 selectively interacts with CCR4+FOXP3+CD4+ T-cells, resulting in preferential transmission of HTLV-1 to Treg cells [6]. This suppressive CD4+CD25+CCR4+ T-cell population is the predominant viral reservoir of HTLV-1-associated ATL [102, 112]. In HAM/TSP patients, the Treg cell subset becomes Th1-like cells that overproduce IFN-*γ*. This is related to leukemia development and it promotes and maintains the FOXP3+ Treg phenotype in ATLL patients [6]. Moreover, other results described an increased regulatory phenotype such as CD39+CCR4+CD25+ FOXP3+CD4+ T-cells in HTLV-1 in infected patients [6, 45]. Hence, HTLV-1 infection can modify FOXP3+ T-cells frequency and phenotype, promoting the central regulator of the host's immune response. Interestingly, other studies reported that a HTLV-1 infection may induce the appearance of iTreg from naïve T-cells involved in the suppression of the viral immune response [118, 123]. Taken together, these findings support the hypothesis that HTLV-1 is one of the exogenous retroviruses responsible for immune disruption along with the increase of FOXP3+ Treg frequency and phenotype. Therefore, HTLV-1 has acquired suitable strategies to achieve persistent infection and favor disease development up to ATLL outbreak in particular by its interactions with Treg cells.

## 4. Role of Treg Cells in the Inflammation Associated with EBV-Induced Nasopharyngeal Carcinoma and Hodgkin's Lymphoma

Epstein-Barr virus (EBV) is one of the most common human viruses, infecting more than 90% of the world's adult population. EBV is a lymphotropic *γ*-herpesvirus whose infection is associated with the development of several tumorous pathologies with a lymphoid or epithelial origin [124–128]. Although EBV is ubiquitous in the general population, in the majority of individuals it persists without causing any diseases. Nevertheless, in some individuals EBV has been implicated in the development of a wide range of cancers. The evidence of a link between EBV and nasopharyngeal carcinoma (NPC) and even Hodgkin's lymphoma (HL) is very strong and both are associated with EBV latency II profile [129].

NPC is an epithelial tumor of the head and neck associated with EBV in almost all cases [130, 131]. NPC is rare in Western countries but endemic in Southeast Asia and in North Africa with an incidence of up to 30–40 cases per 100,000 people in the region of Canton in Southeast China, this suggests a multifactorial etiology of NPC involving EBV infection, genetic predisposition, environmental factors, and other factors still unknown [132–137]. EBV plays a crucial role in NPC development but the comprehension of “when and how” the virus infects epithelial cells is still unsolved. Nevertheless we clearly know that the oral cavity is the gateway of the virus and that it spreads via oropharyngeal secretions and we also know that all NPC tumor cells bear EBV monoclonal viral genome [138–140]. Moreover, EBV is present in the neoplastic cells of approximately 40% of patients with HL, which is characterized by the presence of the malignant Hodgkin's and Reed Sternberg (HRS) cells. These cells only constitute a small part of the entire tumor and are surrounded by a rich background of T- and B-cells, macrophages, and other inflammatory cells [141]. EBV can be detected in the HRS cells (HL) and in NPC cells, in which the virus expresses a limited subset of viral genes including the Epstein Barr nuclear antigen (EBNA)1 and the latent membrane proteins (LMP)1 and LMP2 [142]. Leukocyte infiltration, contrary to an anticipated immune-protective role, could contribute to tumor development and cancer progression. Several studies support the idea of local immune suppression that could explain the lack of efficiency of immune effector cells even after a correct homing.

An important biologic feature of NPC is the presence of a massive lymphoid infiltrate in the primary tumor, which is likely favored by inflammatory cytokines produced by malignant NPC cells [143–146]. It has been shown that most of tumor infiltrating leucocytes (TIL) are CD3+ T-cells with a small resting lymphocytes morphology [147]. Among the CD3+cells, CD4, and CD8 T-cells are found in different proportions depending on the tumor specimens [148]. NK cells were also detected (around 5% of TIL) and small proportions of B-cells are also consistently detected [149, 150]. Dendritic cells are often found inside malignant cell nests whereas monocytes are more often interspersed at some distance of epithelial cell clusters [151]. Eosinophils are also detected in the leukocyte infiltrate of NPC tumors [152]. The leucocyte infiltrate consistently accounts for about 50% of the tumor mass and malignant epithelial cells play an active role in the formation of the infiltrate. Busson's group reported that NPC cells constitutively produce interleukin 1 alpha (IL1*α*), an inflammatory cytokine [143], and this was confirmed by Huang and collaborators. Indeed, they detected IL1*α* and *β* transcripts in most NPC primary tumors, in metastatic lesions, and its absence in control fragments of nonmalignant nasopharyngeal mucosa [144]. IL1*β* was recently described in the recruitment of neutrophils in NPC tumor and it is associated with a better survival rate of patients [153]. Moreover, the inflammatory cytokine IL18, which has structural similarities with IL1, was shown to be consistently produced by malignant NPC cells but not by epithelial cells of the nonmalignant mucosa [154]. Other studies focused on chemokines expression in NPC by showing a consistent expression of CXCL10 by malignant NPC cells. It is known that CXCL10 induces chemotaxis of activated T-cells through its interaction with the CXCR3 receptor. CXCR3 chemoreceptor is associated with Th1 differentiation and is detected in a fraction of T-cells infiltrating the NPC [146].

The expression of several inflammatory cytokines and cell-surface regulatory molecules in primary HRS cells and HL-derived cell lines has been investigated and the result shows that these cells especially produce IL1, 2, 5, 6, 7, 8, 13, 17, TNF*α*, TGF*β*, and CCL28. Through the expression of IL5, CCL28, and TNF*α*, HRS cells are involved in the recruitment of eosinophils that constitute part of the inflammatory infiltrate. IL5 is thought to play a role in eosinophil's differentiation and proliferation [155–158] while TNF*α* was described to be protumorigenic or antitumorigenic depending on the type of intracellular complex formed. The TNF*α*'s impact on cancer also depends on the intracellular signal and thus is triggered in response to TNF*α* binding to its cell-surface receptor [159, 160]. The expression of IL6 and IL7 by HRS cells was involved in the growth and differentiation of B- and T-cells, respectively [158, 161–163]. Moreover, it was reported that HRS cells express both IL6 and IL13 as well as their receptors suggesting that these cytokines may act as potential autocrine growth factors for HRS cells [163–166]. HRS cells also express a number of cytokine receptors and growth factors including IL2R, IL3R, IL4R, IL6R, IL9R, IL13R, CCR7, and CXCR4 [161, 167–169]. Many ligands of these receptors are produced by the inflammatory cells of HL, thus enabling a cooperative crosstalk to take place between HRS cells and the reactive infiltrate. Otherwise, the HL-TIL were shown to express several cytokines such as IL8, IL12, CCR4, and TGF*β*. For example, TIL express IL12 in most cases of HL, particularly in EBV-positive cases [170]. IL12 is important for Th1 differentiation while CCR4 expression contributes to Th2 recruitment [171].

Regarding the immunosuppressive cytokine IL10 in NPC microenvironment, three groups detected it in malignant cells using immunohistochemistry [172–174]. In contrast, Beck's group has failed to detect IL10 transcripts by in situ hybridization in malignant NPC cells [175]. Furthermore, it is important to consider the local immunosuppression; the presence of abnormal quantities of Treg within the tumor site and peripheral blood is a clear indication of this immune suppression [176]. One important indication of the immune evasion in EBV-associated malignancies such as NPC and HL is the lack of immunogenicity of LMP1 antigen. This is an argument in favor of Treg increased activity in case of persistent viral infections. Marshall and collaborators suggested that LMP1 could increase Treg activity and develop this theory (2003). Indeed, they analyzed derived HL patients CD4+ T-cells and reported high levels of IL10 secreting regulatory T-cells (Tr1) associated with a high immunosuppressive activity in HL-TIL. Tr1 suppressive activity is essentially mediated by an IL10 secreting mechanism. Nevertheless, the same group revealed that the immune response was inhibited by a cell-to-cell contact-dependent mechanism; in fact, they described that regulatory activity was avoided by CTLA4 blocking [177].

Moreover, Khanna's group studied Treg capacity to suppress EBV latency antigen response in HL and showed that high expression levels of LAG3, which is a Treg marker [178], is associated with a LMP1/2 specific T-cells function loss within HL-TIL. They also showed regulatory properties in CD4+LAG3+ T-cells [179]. They have shown an association between LAG3 and EBV gene expression in tumorous tissues and that LAG3 was more frequently expressed than FOXP3 in lymphocytes. Those cells can only express one of the two markers. In peripheral blood, CD4+LAG3+ T lymphocytes were enriched with CTLA4 and GITR but not FOXP3; this phenotype was more frequent in patients with an active disease than those with an inactive one [179].

In order to understand Treg specificity in EBV infection, Voo and collaborators stimulated PBMC* in vitro* using EBNA1 peptides. They indicated that T-cells expansion included not only helper CD4+ T clones but also regulatory CD4+ T-cells which exhibit simultaneous regulatory phenotype and effective suppressive activity [180]; both cell types recognize the same EBNA1 epitopes. Moreover, the suppressive mechanism is cell-to-cell contact-dependent, although some clones remain capable of suppression even in transwell assays; this shows a soluble inhibition which was neither IL10 nor TGF*β* [180]. Nevertheless, we must consider the Treg/T helper ratio and verify Treg expansion when using peptide vaccine in order to treat EBV associated diseases since this could have serious consequences on patients.

Marshall's group showed the immunosuppressive properties of HL-TIL and the capacity of LMP1 to stimulate Treg in healthy donors; then they suggested that LMP1 is involved in the Treg's increased activity in HL. They compared Th1, Th2, and Treg responses to LMP1 within PBMC and TIL in EBV-negative and -positive HL patients. EBV-positive HL patients presented,* ex vivo,* an increased number of both IL10-secreting and CTLA4+ cells comparing to EBV-negative HL patients in periphery and in lymph nodes. In most patients, both PBMC and TIL responses to LMP1 were characterized by IL10 secretion [177, 181]; this highlights the crucial role of IL10-secreting cells in favoring tumor immune evasion in EBV-positive HL patients.

Moreover, Treg are constantly detected in NPC biopsies. Lau et al. showed a significant expansion of CD4+CD25^high^ T-cells in 56 NPC patients comparing to healthy controls. They reported an average of 12% of CD4+CD25^high^FOXP3+ T-cells within TIL and an average of 8,2% of CD4+CD25^high^ T-cells within circulating T-cells [176]. This high Treg frequency in NPC patients may partly compromise the chance of success of immunotherapy strategies targeting T-cells.

Very recently, we found that tumor NPC exosomes, which are nanoparticles that have immunosuppressive properties, interact with Treg, participate in their massive recruitment into the tumor, and enhance their suppressive activity. We also showed that NPC exosomes promote CD4+CD25− T-cells conversion into iTreg. This conversion was accompanied by a dose-dependent TGF*β* secretion [48]. CCL20 or MIP-3*α* is a chemokine that induces leukocyte migration into the inflammation site and regulates leukocyte trafficking through lymphoid tissues. Chan's group detected high CCL20 concentrations in serum samples from NPC patients [182]. We also showed for the first time, using both Western Blot and electron microscopy, the presence of CCL20 on exosomes and we demonstrated that it plays a crucial role in Treg recruitment into the NPC tumor microenvironment [48].

Although EBV-specific cytotoxic T-cells (CTLs) can be detected in HL patients and have been shown to kill LMP1 and LMP2 expressing cells* in vitro*, they are unable to eliminate EBV-infected tumor cells* in vivo* [183]. Several hypotheses have been made to explain the apparent inefficiency of the antitumoral immune response in HL patients. Some studies suggested that CD4+ T lymphocytes producing Th2 cytokines and chemokines could probably contribute to the local immunosuppression of Th1 cellular immune response [167, 184]. Others explain this failure by an increase in the recruitment of Treg [185–187].

It has been shown that the percentage of CD4+CD25+ nTreg is elevated in the peripheral blood of HL patients compared to healthy donors, as well as in the blood of patients having an active disease compared to those in remission [179, 188]. Their number is also increased in HL tumor tissues where they are found in close proximity to HRS cells [177, 188, 189]. In addition, our group has recently pointed out that Tr1 was strongly recruited in HL patients. Indeed, it has been shown that an important increase of Treg and Tr1 marker genes (CD4, CD25, FOXP3, GITR, CTLA4, CD49b, CD18, P-Selectin, LAG3, and CD40L) expression levels in EBV-positive HL nodes compared to EBV-negative and control nodes. A similar increase was observed within immunosuppressive cytokine genes (IL10 and TGF*β*) expression and their receptors (IL10R*α*, IL10R*β*, and TGF*β*RII). Furthermore, an upregulation was also confirmed for the gene expression of several chemokines (CCL4, CCL5, CCL17, CCL22, CCL20, and CXCL9), known to strongly attract Treg and their receptors (CCR4, CCR5, and CCR7). Moreover, we confirmed an increase of Tr1 cells in EBV-positive HL patients by determining CD4, CD49b, CD18, and also IL10 protein levels in nodes and biopsies [190].

The role of Treg in EBV infection is interesting for many reasons and the elucidation of its role in primary EBV infection could facilitate the immunomanipulation at initial infection stages in order to limit infectious mononucleosis symptoms and prevent subsequent HL or NPC risk. Treg frequency and functional capacity could affect viral persistence level and then, the clarification of Treg role could partly explain EBV-associated tumor progression and attain the development of new therapeutic tools.

## 5. Conclusion

As discussed in this review, there is critical evidence for a strong relation between inflammation and cancer development. As noted above, HCV-, HLTV-1-, and EBV-infections activate the immune system's response involving a wide range of cellular components. The main goal of this immune response is to eradicate the pathogen mainly through an inflammation mechanism. The inflammation process involves the release of several cytokines and chemokines by various proinflammatory cells or by cancer cells themselves. The release of proinflammatory factors by such cells leads to the recruitment of numerous cells to the infection site. Numbers of these cells consist of Treg cells which control inflammation and avoid chronic inflammation. In this review, it has been reported that regulatory CD4+ T-cells were associated with a poor prognosis in HCC, ATLL, and NPC patients. It was also reported that HTLV-1, HCV, and EBV viruses increase the frequency and the activity of regulatory T-cells in infected patients. Both HCV and HTLV-1 were able to hijack the immune system, thus favoring the viral persistence. In addition, a high frequency of Tr1 cells was reported in Hodgkin Lymphoma and HCC patients with a high suppressive activity mediated through IL10. In HCV-related diseases, as well as in HTLV1- and EBV-related diseases, regulatory T-cells overexpressed several cell surface markers and thereby, increase the cell-to-cell dependent suppressive mechanism. We also mentioned the important recruitment of regulatory nTreg and iTreg within HCC and NPC tumors. Otherwise, others results reported that EBV-associated NPC exosomes were able to induce the conversion of CD4+CD25− T-cells into nTreg. Similarly, there was some evidence of antigen specific conversion of CD4+ T-cells into iTreg (Th3 and Tr1) after HCV infection. Taken together, these data strongly suggest that the frequency of Treg is proportionally correlated to the aggravation of the disease. Nevertheless, there are some noticeable differences in the involvement of Treg cells in these diseases. First, it is well known that HCV and HBV viruses are essentially hepatotropic even if some recent studies have suggested a possible lymphotropism. In contrast, natural regulatory CD4+CD25+FOXP3+ T-cells have been specifically described as the main HTLV1 reservoir. This close interaction between Treg and HTLV-1 results in preferential transmission of HTLV-1 to Treg cells promoting the central regulator of the host immune response* in vivo*. Secondly, the Treg suppressive activity in HCV infection passed mainly through cytokine production while in HTLV-1 infection, it was mainly through a cell-to-cell dependent mechanism. Others reported results suggesting that ATLL regulatory T-cells may lose their suppressive function with time but no studies have reported this in HCC, nor in NPC. In all cases, regulatory T-cells can directly inhibit the effector cells proliferation, cytotoxic function, cytokine production, and antiviral response. These regulatory cells produce high amounts of IL10 and TGF*β*, promoting the virus to achieve persistent infection. These viruses can increase both the frequency and phenotype of Treg cells and can therefore increase their suppressive function. Finally, these findings describe how viruses develop intelligent strategies to achieve persistent infection and, sometimes, the development of tumors. Viruses may directly induce dysfunctions of the host's immune response to increase their pathogenicity and then, the regulatory mechanisms of the immune inflammatory response are of major importance.
